# Paediatric sedation with intranasal dexmedetomidine: Protocol for a systematic review and meta-analysis

**DOI:** 10.1371/journal.pone.0317406

**Published:** 2025-01-13

**Authors:** Katrine Dueholm Nissen, Caroline Margaret Moos, Andras Wolf, Thomas Strøm

**Affiliations:** 1 Department of Anaesthesiology and Intensive Care, University Hospital of Southern Denmark, Aabenraa, Denmark; 2 Department of Clinical Research, University Hospital of Southern Denmark, Aabenraa, Denmark; 3 Anaesthesiology and Intensive Care Research Unit, Department of Regional Health Research, University of Southern Denmark, Aabenraa, Denmark; Sapienza University of Rome: Universita degli Studi di Roma La Sapienza, ITALY

## Abstract

**Introduction:**

Sedation ensures a child remains motionless during a procedure and decreases anxiety. Several pharmacologic regimes exist for paediatric sedation. However, often, intravenous cannulation is required, causing distress for the child. Creating a low-stress environment for children during medical procedures is crucial. Intranasal dexmedetomidine offers a promising alternative by either removing the need for intravenous cannulation or significantly reducing stress and anxiety when cannulation is necessary. We aim to investigate the safety and efficiency of sedating children with intranasal dexmedetomidine.

**Methods and analysis:**

We will systematically search MEDLINE (Ovid), Embase (Ovid), CINAHL (EBSCO), CENTRAL, Clinicaltrials.gov, and the WHO ICTRP portal. We will include all randomized controlled trials (RCT) that investigate the use of intranasal dexmedetomidine compared to alternative sedatives for premedication or sedation of children. Two researchers will independently screen title/abstract and full-text articles for eligibility using Covidence. Our primary outcome is sedation success rate. RCTs that meet the inclusion criteria will form the unit of analysis. Data extracted from each study will be presented in table format (S2 Table). Information on parameters that describe safety and efficiency outcomes will be extracted and analysed. Outcome data will be reported as risk ratios and 95% confidence intervals (CI) for dichotomous outcomes or mean and standardized mean differences with 95% CI for continuous outcomes. The assessment of statistical heterogeneity will be examined using Chi^2^- and I^2^-statistics. PROSPERO registration number CRD42024532993

**Discussion:**

Sedation with intranasal dexmedetomidine is not common practice in all countries, though the medicament has the potential to provide a child-friendly approach to sedation and premedication. Reviews on the area are conflicting, and new RCT studies have been published. Our systematic review aims to comprehensively assess intranasal paediatric sedation, focusing on dexmedetomidine and guiding clinicians in daily decision-making for optimal paediatric sedation.

## Introduction

Children encounter challenging situations during a hospital stay. The trauma of illness can be exacerbated by the testing that is required to make a diagnosis. Some children may require a magnetic resonance imaging (MRI) scan, stipulating that the child must lie completely still for a significant amount of time. Other children need intravenous access or a mask induction before general anaesthesia in order to complete a medical procedure. A successful child-friendly approach to paediatric sedation is medication and techniques that minimize pain and anxiety associated with unpleasant medical procedures [1].

Paediatric sedation can be accomplished by administering oral, rectal, intravenous, or intranasal medicine [2, 3]. Paediatric sedation may involve multiple sedatives or premedication strategies, as there is no ideal strategy [3]. However, children have described peripheral intravenous catheter insertion as the most painful and stressful procedure during their hospitalization [1, 4]. Therefore, propofol, which only can be administered intravenously, is not ideal. Midazolam has an unpleasant taste, may cause nasal irritation, and comes with a risk of a paradox reaction. Ketamine can potentially induce a dissociative state, and the alpha-2-receptor agonist clonidine requires approximately 45–60 minutes to take effect [3, 5]. Therefore, paediatric sedation can be challenging to accomplish without compromising the child-friendly approach.

Dexmedetomidine is a common agent used for sedation in intensive care units. Recently, it has been introduced as a sedative agent for the paediatric population [6, 7] and may be the optimal choice. Dexmedetomidine is an alpha-2-receptor agonist with a seven to eight times higher affinity with the receptor than clonidine [8]. Dexmedetomidine is administered intranasally, has no smell or taste, does not cause nasal irritation, has a relatively short onset time and appears to provide sedation and analgesia with minimal side effects [6, 7, 9]. Sedation with dexmedetomidine produces a cooperative and rousable sedative effect [7] and has been shown to elicit electroencephalogram activity similar to natural sleep [10]. As a child-friendly sedative regime focuses on the child being comfortable and cooperative, it is worthwhile exploring the evidence base for using intranasal dexmedetomidine for the sedation of children.

The implementation of intranasal dexmedetomidine for paediatric sedation is well-established in some countries [11, 12]. Sedation with intranasal dexmedetomidine has been investigated in randomized controlled trials (RCT) for MRI and computerized tomography (CT) scans [13, 14], eye examination [15], transthoracic echocardiogram (TTE) [16], dental work [17], auditory brainstem response (ABR) [18], laceration repair [19] and as premedication to accept either mask induction or intravenous insertion before general anaesthesia [20, 21]. Some systematic reviews have reported intranasal dexmedetomidine’s general safety and efficiency in paediatric sedation [22–27]. These reviews focus primarily on comparing dexmedetomidine to one alternative sedative or with a specific investigation or procedure. Furthermore, only a few systematic reviews have focused exclusively on including RCTs. For example, a recent review [25] included 19 RCTs with various procedures but investigated only two different sedatives with dexmedetomidine. Since the publication, additional RCTs have been completed [28–32].

Although some reviews [24, 27, 33, 34] conclude that intranasal dexmedetomidine was effective and safe, these reviews included cohort studies or only looked at a single other sedative or procedure. De Rover [26] suggested that dexmedetomidine as a solo sedative may not be sufficient for MRI scans, but Lyu [22] disagreed with this conclusion.

Therefore, we aim to synthesize information from all RCT studies examining the safety and efficiency of intranasal dexmedetomidine for children requiring sedation. Moreover, we are interested in identifying areas where further research into dexmedetomidine and paediatric sedation is required.

## Methods

This study protocol has been registered in the International Prospective Register of Systematic Reviews (PROSPERO) (registration number CRD42024532993). This review will be guided by the Cochrane’s Handbook for Systematic Reviews of Interventions [35]. The Preferred Reporting Items for Systematic Reviews and Meta-analysis (PRISMA) will be adhered to [36]. To conduct this protocol, we adhered to the PRISMA-P reporting guidelines for protocols (S1 Checklist) [37].

### Study design

We will only consider peer-reviewed RCTs. A study will qualify as an RCT if it is a prospective study where patients were randomly allocated to one or more interventions or a control group. Observational or retrospective studies will be excluded.

### Eligibility criteria

The eligibility criteria for this review have been developed using PICO typology (population, intervention, comparison, and outcomes) and are described below. The online databases will be searched from their inception to the date of our final search, without any time restrictions applied. RCTs will be included if they meet the following criteria.

### Participants

Children aged between 0–17 years sedated with intranasal dexmedetomidine. There will be no limitation on the American Society of Anaesthesiologists (ASA) physical status classification. All procedures or scanning modalities that investigate children sedated with intranasal dexmedetomidine will be considered, regardless of the length of examination or the painfulness of the procedure. We define procedures as interventions to diagnose, measure, or examine the child. Studies using intranasal dexmedetomidine as premedication to facilitate the acceptance of mask induction and intravenous cannulation before general anaesthesia will also be included Outcomes from populations with mixed ages where children cannot be isolated will be excluded.

### Interventions/comparators

The intervention group are paediatric patients sedated with intranasal dexmedetomidine at any dose for any intervention. The control group will be any other sedative, independent of the route of administration. Studies that describe a control group that combines several sedatives will also be included but excluded if the control group also includes dexmedetomidine.

### Outcome measures

The primary outcomes of interest are:

- Sedation success rate. We define successful sedation as sedation to the extent that the examination, procedure, intervention or scan can be completed successfully with intranasal dexmedetomidine. For example success rate with intranasal dexmedetomidine for interventions such as a) mask induction using inhalation anaesthetics or b) intravenous cannulation prior to general anaesthesia. We will use each RCT’s own definition of sedation success, regardless of whether it is based on a formal tool or a subjective assessment. By incorporating the definitions provided within each study, we aim to evaluate sedation success in the context of intranasal dexmedetomidine without restricting the methods or criteria used.

Additional outcomes include:

- Prevalence of adverse effects. Adverse events are defined as hypotension, bradycardia, hypertension, nausea, vomiting, apnea, laryngospasm or de-saturation requiring intervention as described by Bhatt et al. [38]. If studies fail to report adverse events described by Bhatt, we will present the data on adverse events as they are described in the included studies.
- Onset time of sedation, defined as the time between administration of sedative until time of adequate sedation level
- Sedation duration is defined as the time between adequate sedation level until the patient is fully awake and alert
- Depth of sedation defined by a classification tool such as ramsay sedation scale, sedation state scale, modified observers assessment of alterness/sedation scale (MOAAS) or University of Michigan sedation scale to describe the adequacy of the sedation.
- Recovery time defined as the time between fully awake and alert and hospital discharge
- Need for additional sedation
- Post-sedation agitation
- Concentrations, dosage, and technique of intranasal dexmedetomidine application.

### Search strategy

We will search for RCTs from the following databases and registries: MEDLINE (Ovid), Embase (Ovid), CINAHL (EBSCO), US National Institutes of Health Ongoing Trials Register (Clinicaltrials.gov), International Clinical Trials Registry Platform (ICTRP) and the Cochrane Central Register of Controlled Trials (CENTRAL). There will be no language restrictions. If studies are written in languages other than English or Danish, we will seek help with translation. We will perform a block search using selected search terms. The search will combine three blocks i) dexmedetomidine, ii) RCT and iii) intranasal. Each block will include medical subject headings (MeSH) and free text words. The formation of the search block for dexmedetomidine was inspired by a recent Cochrane review [39]. A medical information specialist has reviewed and adjusted the search strategy. The search strategy for Medline can be found in the supplementary material (S1 Table). The Web of Science database will be used for forward and backward citation searches of the included studies to identify any additional references. In addition, relevant reviews will also be searched for studies to identify studies eligible for inclusion.

Two researchers will independently screen title/abstract and full-text articles for eligibility using Covidence, a web-based collaboration software platform that streamlines the production of systematic and other literature reviews and removes duplicates. Any articles excluded at full-text screening will be tagged with a reason for exclusion. A third reviewer will reconcile disagreements. If data from a study is missing, authors will be contacted as necessary.

### Data extraction

We will present the information extracted from each study in a tabular format. This will include data such as i) author/year, participants (age, The American Society of Anesthesiologists (ASA) physical status classification status, sex, weight, setting, intervention, and the number of participants), ii) intranasal dexmedetomidine (dose and frequency), iii) the comparison (drug, dose, frequency, route of administration) and iv) assessed outcomes. Two authors will independently extract all data and resolve potential conflicts through discussion.

### Data synthesis and analysis

Any RCT that meets the inclusion criteria will form the unit of analysis. We will calculate risk ratios with 95% confidence intervals (CI) for dichotomous outcomes. We will calculate the mean or standardized mean difference and 95% CI for continuous outcomes. The assessment of statistical heterogeneity will be examined using Chi^2^- and I^2^-statistics in relation to variance between studies [35, 40, 41]. We will consider I^2^ values >50% as evidence of heterogeneity [35]. If there is substantial heterogeneity, we will explore the source by analyzing the change in I^2^ in the stratified analysis. A meta-analysis will be performed, and forest plots will illustrate the results. Depending on the number of studies and degree of heterogeneity, a subgroup analysis on the sedation comparator and procedural comparators will be performed. Furthermore, we will perform subgroup analysis separating painful and non-painful procedures in our analysis. If the data is too heterogeneous, we will provide a narrative synthesis. The analysis will be conducted using Stata (latest version available, StataCorp LLC, College Station, TX).

### Risk of bias assessment and certainty of evidence

Two researchers will independently assess the risk of bias for each included study using the Cochrane risk of bias tool (Rob 2) [42]. We will assess the certainty of the evidence using the Grading of Recommendations Assessment, Development, and Evaluation (GRADE).

## Discussion

The use of intranasal dexmedetomidine in paediatric sedation is not common practice in Denmark. However, it is well-known in other Scandinavian countries, such as Sweden and Norway [12]. So why are Danish clinicians hesitant to change practice? Although the evidence on intranasal dexmedetomidine is comprehensive, it is also conflicting. The recent review from 2020 [25] included 19 RCTs but did not include RCTs in which intranasal dexmedetomidine is used as premedication. Moreover, additional RCTs on intranasal dexmedetomidine for the paediatric population have since been published. A systematic review of all paediatric sedation and premedication in relation to intranasal dexmedetomidine is preferable to be transferable in a clinical setting. Clinicians specialized in caring for paediatric patients often encounter different clinical settings. A review that synthesizes the evidence about how intranasal dexmedetomidine can be used for various interventions across differing age groups enables clinicians to reflect on their daily, individualised practice.

Child development and the inherent changes between birth and adolescence can make general sedation strategies for the paediatric population challenging. We are convinced that a systematic review considering intranasal dexmedetomidine in a broader context will help clinicians choose the best sedation option for specific age groups or procedures to reduce stress and anxiety.

## Supporting information

S1 Checklist PRISMA-P. (DOCX)

S1 Table Search performed in MEDLINE. (DOCX)

S2 Table Suggested table of data collection. (DOCX)
