# Supplementary material for: Paediatric sedation with intranasal dexmedetomidine: Protocol for a systematic review and meta-analysis
Source: PLoS One. 2025 Jan 13;20(1):e0317406. doi: 10.1371/journal.pone.0317406 (PMC11729917; doi:10.1371/journal.pone.0317406)
Supplement: S1 Checklist — (DOCX) [file pone.0317406.s001.docx]

**S1 CHECKLIST PRISMA-P**

**PRISMA-P (Preferred Reporting Items for Systematic review and Meta-analysis Protocols) 2015 checklist: recommended items to address in a systematic review protocol***

| Section and topic | Item No | Checklist item |
| --- | --- | --- |
| ADMINISTRATIVE INFORMATION | | |
| Title: |  |  |
| Identification | 1a | Paediatric sedation with intranasal dexmedetomidine: a systematic review protocol (title page) |
| Update | 1b | N/A |
| Registration | 2 | PROSPERO registration number CRD42024532993 |
| Authors: |  |  |
| Contact | 3a | All names, institutional affiliation, e-mail address of all protocol authors (title page and application form) |
| Contributions | 3b | KDN and CMM drafted the manuscript. AW provided expert knowledge. All authors read, provided feedback and approved the final manuscript. TS is the guarantor. |
| Amendments | 4 | The protocol does not represent an amendment of previously work. |
| Support: |  |  |
| Sources | 5a | No funding or sponsorship has been provided for this review |
| Sponsor | 5b | N/A |
| Role of sponsor or funder | 5c | N/A |
| INTRODUCTION | | |
| Rationale | 6 | The rationale for the review in the context of what is already known is described in manuscript (Page 3-4) |
| Objectives | 7 | We provide an explicit statement of the questions the review will address (Page 4). This review protocol has been developed using PICO (participants, interventions, comparators and outcomes) (Page 5) |
| METHODS | | |
| Eligibility criteria | 8 | Eligibility criteria are described (Page 5-6) |
| Information sources | 9 | We describe our search strategy and databases that will be used (Page 7-8) |
| Search strategy | 10 | A draft of our search strategy in included in our protocol (S1 Appendix) |
| Study records: |  |  |
| Data management | 11a | We describe the mechanisms that will be used to manage records and data throughout the review (Page 8) |
| Selection process | 11b | We describe the process that will be used for selecting studies through each phase of the review (Page 7-8) |
| Data collection process | 11c | We describe planned method of extracting data from reports (Page 8) |
| Data items | 12 | We list and define all variables for which data will be sought (Page 8-9) |
| Outcomes and prioritization | 13 | We list and define all outcomes for which data will be sought (Page 6-7) |
| Risk of bias in individual studies | 14 | We describe anticipated methods for assessing risk of bias of systematic reviews (Page 9) |
| Data synthesis | 15a | We describe criteria under which study data will be quantitatively synthesised (Page 8-9) |
|  | 15b | We describe planned summary measures, methods of handling data and methods of combining data from studies, including any planned exploration of consistency (such as I^2^) (Page 8-9) |
|  | 15c | We describe any proposed additional analyses (such as sensitivity or subgroup analyses, meta-regression) (Page 8-9) |
|  | 15d | If quantitative synthesis is not appropriate, a narrative synthesis will be performed (Page 8-9) |
| Meta-bias(es) | 16 | We specify planned assessment of meta-bias(es) (such as publication bias across studies, selective reporting within studies) (Page 8-9) |
| Confidence in cumulative evidence | 17 | We describe how the strength of the body of evidence will be assessed with GRADE (Page 9) |

*** It is strongly recommended that this checklist be read in conjunction with the PRISMA-P Explanation and Elaboration (cite when available) for important clarification on the items. Amendments to a review protocol should be tracked and dated. The copyright for PRISMA-P (including checklist) is held by the PRISMA-P Group and is distributed under a Creative Commons Attribution Licence 4.0.**

*From: Shamseer L, Moher D, Clarke M, Ghersi D, Liberati A, Petticrew M, Shekelle P, Stewart L, PRISMA-P Group. Preferred reporting items for systematic review and meta-analysis protocols (PRISMA-P) 2015: elaboration and explanation. BMJ. 2015 Jan 2;349(jan02 1):g7647.*
