# Supplementary material for: Paediatric sedation with intranasal dexmedetomidine: Protocol for a systematic review and meta-analysis
Source: PLoS One. 2025 Jan 13;20(1):e0317406. doi: 10.1371/journal.pone.0317406 (PMC11729917; doi:10.1371/journal.pone.0317406)
Supplement: S1 Table — (DOCX) [file pone.0317406.s002.docx]

## S1 Table: Search performed in MEDLINE Ovid MEDLINE(R) ALL <1946 to May 24, 2024>

| **#** | **Query** | **Results from 26 May 2024** |
| --- | --- | --- |
| 1 | dexmedetomidine/ | 5,765 |
| 2 | (dexmedetomidin* or bxcl 501 or bxcl501 or cepedex or da 9501 or da9501 or dexdor or mpv 1440 or mpv1440 or precedex or primadex or sedadex or sileo or "tpu 006" or tpu006).ti,ab,kw,kf. | 9,361 |
| 3 | 1 or 2 | 9,600 |
| 4 | exp randomized controlled trial/ | 615,302 |
| 5 | controlled clinical trial.pt. | 95,537 |
| 6 | randomized.ab. | 646,772 |
| 7 | placebo.ab. | 248,568 |
| 8 | drug therapy.fs. | 2,697,780 |
| 9 | randomly.ab. | 434,066 |
| 10 | trial.ab. | 699,204 |
| 11 | groups.ab. | 2,682,369 |
| 12 | 4 or 5 or 6 or 7 or 8 or 9 or 10 or 11 | 5,967,770 |
| 13 | exp animals/ not humans.sh. | 5,224,753 |
| 14 | 12 not 13 | 5,221,022 |
| 15 | 3 and 14 | 4,558 |
| 16 | exp Administration, Intranasal/ | 16,690 |
| 17 | exp "Nebulizers and Vaporizers"/ | 13,039 |
| 18 | exp Administration, Topical/ | 96,984 |
| 19 | (spray or aerosol or powder or inhal* or solution or turbuhaler or intranasal* or intra next nasal or topical* or drops).tw. | 1,067,890 |
| 20 | 16 or 17 or 18 or 19 | 1,116,518 |
| 21 | 3 and 14 and 20 | 509 |
