# Supplementary material for: Paediatric sedation with intranasal dexmedetomidine: Protocol for a systematic review and meta-analysis
Source: PLoS One. 2025 Jan 13;20(1):e0317406. doi: 10.1371/journal.pone.0317406 (PMC11729917; doi:10.1371/journal.pone.0317406)
Supplement: S2 Table — (DOCX) [file pone.0317406.s003.docx]

## S2: Suggested table of data collection

| **Primary study (Author, year, country)** | **Participants (Age, weight, number of participants)** | **Examinations/procedures** | **Intervention/Dexmedetomidine (Dose, frequency)** | **Comparator (Drug, dose, frequency)** | **Outcome measures** | **Effect size** | **Quality assessment** |
| --- | --- | --- | --- | --- | --- | --- | --- |
| **Studie 1** |  |  |  |  |  |  |  |
| **Studie 2** |  |  |  |  |  |  |  |
| **…** |  |  |  |  |  |  |  |
